# The Longitudinal Association Between Physical Activity and Physical Function in Older Women

**DOI:** 10.3389/fspor.2022.879025

**Published:** 2022-07-22

**Authors:** Christie L. Ward-Ritacco, Mary L. Greaney, Phillip G. Clark, Deborah Riebe

**Affiliations:** ^1^Department of Kinesiology, University of Rhode Island, Kingston, RI, United States; ^2^Department of Health Studies, University of Rhode Island, Kingston, RI, United States; ^3^Program in Gerontology and Rhode Island Geriatric Education Center, University of Rhode Island, Kingston, RI, United States

**Keywords:** physical activity, older women, physical function, transtheoretical model, Timed Up and Go test

## Abstract

**Purpose:**

The age-related decline in physical function is ameliorated by physical activity; however, less is known about changes in physical function in active vs. inactive older women. The purpose of this study was to determine the longitudinal associations between physical activity and physical function in community-dwelling older women.

**Methods:**

238 participants (age 79.0 ± 5.1) were dichotomized into two activity groups [inactive (IG); *n* = 144 or active (AG); *n* = 94] based on self-reported exercise at baseline. Repeated measures ANCOVA, controlling for age, measured differences in physical function between activity groups at baseline and 48-months using the Timed Up and Go, 30-s chair stand, and 30-second arm curl. Differences in Timed Up and Go classification [normal (≤ 8.23 s); preclinical limitations/limited physical function (>8.23 s)] were analyzed using chi-square tests for activity group and for activity-age group (AG, <80 years; AG, ≥ 80 years; IG, <80 years; IG, ≥ 80 years).

**Results:**

The repeated measures ANCOVA yielded a significant main effect for activity group for the Timed Up and Go (*p* = 0.006), 30-s chair stand (*p* = 0.002) and 30 s arm curl (*p* = 0.007) and a significant time main effect for the Timed Up and Go (*p* = 0.016). There were no significant group by time interactions. A larger proportion of the IG than the AG (58.2 vs. 86.5%, respectively) had Timed Up and Go scores >8.23 s (*p* < 0.001). At 48-months, individuals in the AG were more likely to have normal Timed Up and Go scores compared to those in the IG in both age groups [$\chi_{\left(3,\;\text{N}=236\right)}^{2}$ = 42.56, *p* < 0.001].

**Conclusion:**

Older women who engaged in regular exercise at baseline had higher levels of objectively measured physical function and were less likely to have abnormal Timed Up and Go scores. These findings help illustrate the long-term benefit of exercise on physical function in older women.

## Introduction

The aging process is accompanied by a decline in physical function (Milanović et al., 2013). Limitations in physical function are associated with several adverse outcomes in older adults, including increased risk of falling (Dipietro et al., 2019), impaired abilities to perform the activities of daily living (Gill et al., 2012), lower quality of life (Musich et al., 2018), hospitalizations (Callahan et al., 2018), nursing home admissions (Gustavson et al., 2019), healthcare expenditures (Musich et al., 2018), and mortality (Pavasini et al., 2016). Between 2015 and 2040, the number of U.S. adults aged 65 and older will increase from 47.8 million to 82.3 million, representing an increase in the proportion of the total population from 13 to 20% (Administration for Community Living U.S. Department of Health and Human Services, 2016). As age is a primary risk factor for physical function decline (Leigh et al., 2017; Metti et al., 2018; Xu et al., 2020), the growing number of older adults will result in a greater proportion of the population experiencing functional impairment.

Compared to men, women have lower levels of physical function and experience a more rapid age-related decline in physical function (Newman et al., 2006; Centers for Disease Control and Prevention, 2009; Straight et al., 2013; Suetta et al., 2019). The Copenhagen Sarcopenia Study examined a large cohort of healthy adults (*n* = 1,305) aged 20–93 years and found sex differences in the 70–80-year-old age group, with men performing a significantly higher number of chair stands in 30 s than women (Suetta et al., 2019). The incidence and prevalence of disability are also higher among women compared to men (Lee et al., 2021). Recently, Lee et al. (2021) found that disability incidence rates for men ranged from 0.4 to 5.0, compared to 0.5–9.4 for women.

A decline in physical function is accentuated by a physically inactive lifestyle (Harridge and Lazarus, 2017). Participation in regular exercise is a widely accepted approach to countering the age-related loss of physical function in older adults (Physical Activity Guidelines Advisory Committee, 2018; Dipietro et al., 2019). For example, Paterson and Warburton (Paterson and Warburton, 2010) found that the relative risk of losing functional independence may decrease up to 30% by participating in 150–180 min of moderate-to-moderately vigorous intensity physical activity each week and an additional 30% if activities are more vigorous in nature. The improvement in physical function is a result of numerous physiological outcomes associated with regular exercise, such as improvements in muscular strength and balance and decreases in percent body fat (Physical Activity Guidelines Advisory Committee, 2018). Despite the known benefits of physical activity for physical function with aging, the proportion of older adults meeting recommended physical activity guidelines remains low (Federal Interagency Forum on Aging-Related Statistics, 2020).

With the rapid aging of the U.S. population, it is important to elucidate the role that exercise plays in preventing and slowing age-related declines in physical function. In addition, since women have greater longevity and higher rates of disability than men (Federal Interagency Forum on Aging-Related Statistics, 2020), there is a particular need to further understand physical function in this population. Therefore, the purpose of this study was to measure the longitudinal associations between physical activity and physical function in older women. A secondary purpose was to examine patterns of change in physical function in active and inactive older women in different age groups (<80 years; ≥80 years).

## Materials and Methods

### Study Design and Participants

This is secondary analysis using data from the Study of Exercise and Nutrition in Older Rhode Islanders (SENIOR) II Project, a 4-year longitudinal randomized controlled trial of an intervention designed to help older adults maintain a physically active lifestyle and fruit and vegetable intake in the face of increasing challenges associated with aging. The SENIOR II Project was a follow-up to the SENIOR Project, a 12-month randomized community-based intervention designed to increase physical activity and fruit and vegetable intake followed by a 12-month non-intervention follow-up period. The SENIOR II Project began ~3.5 years after the completion of the SENIOR Project. A full description of the SENIOR and SENIOR II projects have been previously published (Clark et al., 2011).

To be included in SENIOR II, participants were required to: (1) have participated in the original SENIOR Project; and (2) be in the action or maintenance stage for exercise and/or fruit and vegetable consumption at the end of the SENIOR Project. Participants were excluded from SENIOR II if they had low levels of cognitive function [i.e., had a score below 15 on the Folstein Mini-Mental Examination, MMSE (Folstein et al., 1975)] or if they answered “yes” to one or more medical screening questions and were unable to secure medical clearance. Face-to-face assessments were conducted in the participants' homes or at the research project office located in the City of East Providence by trained research assistants who were also community-dwelling older adults. The research protocol was approved by the University of Rhode Island Institutional Review Board. All participants were fully informed about the study's nature, procedures, and risks and provided their informed consent.

At baseline, The SENIOR II Project had 471 male (*n* = 119) and female (*n* = 352) participants. The current study included only female participants due to the increased risk of physical function decline experienced by aging women compared to men. Of the 352 female participants, 114 dropped out of the study (37.2% died, 23% not interested/too busy, 18.6% developed illness/relocated to nursing home, 7.9% did not have PCP/caregiver approval, 6.2% had cognitive issues, 7.1% other).

To allow for the inclusion of as many participants as possible in the current study, we included all participants with baseline and 48-month data for at least one variable of interest. This resulted in varying samples sizes for each analysis; sample sizes are noted in the results. Of the 238 participants who did not drop out of the SENIOR II Study, 165 participants completed all three assessments of objective physical function (69%), 29 participants (12.2%) are missing baseline or follow-up data for one measure, and 44 participants (18.5%) are missing two or more measures.

Compared to those who dropped out, individuals who remained in the Senior II Project were younger (79.0 ± 5.1 yrs vs. 82.0 ± 6.8 yrs, *p* < 0.001), and had higher body weight (69.5 ± 13.7 kg vs. 65.6 ± 13.6 kg, *p* = 0.013), faster Timed Up and Go scores [11.1 ± 4.9 s (*n* = 231) vs. 15.3 ± 12.8 s (*n* = 109); *p* < 0.001] and a greater number of chair stands [10.6 ± 3.6 reps (*n* = 230) vs. 9.1 ± 4.3 reps (*n* = 101); *p* = 0.002] at baseline. There were no significant differences between those who dropped out and those who remained in the SENIOR II Project for race/ethnicity, years of education attained, BMI, or arm curl repetitions (all *p* >.05).

### Measures

#### Demographics

Sociodemographic and lifestyle factors, including age, race, ethnicity, education, smoking status, and perceived health status (excellent, very good, good, fair, poor) were assessed. Body height was measured to the nearest 0.1 cm using a portable stadiometer. Body weight was measured to the nearest 0.1 kg with a digital floor scale by trained research assistants, and BMI was calculated using standard methods. The first question from the Medical Outcomes Study 36-item Short Form (McHorney et al., 1993) was used to assess perceived health status.

#### Physical Function Measures

##### Timed Up-and-Go

The Timed Up and Go measures, in seconds, the time taken by an individual to stand up from a standard chair, walk a distance of 3-meters, turn, walk back to the chair, and sit down again. Timed Up and Go scores are associated with risk of falls (Alexandre et al., 2012), health status (Viccaro et al., 2011), capacity to perform activities of daily living (ADLs) (Rydwik et al., 2011; Viccaro et al., 2011), and physical function (van Iersel et al., 2008; Rydwik et al., 2011; Viccaro et al., 2011). The Timed Up and Go demonstrates high inter-rater reliability (ICC-0.99), as well as moderate validity (*r* = −0.51 to −0.72) with other measures of physical function in older adults (Podsiadlo and Richardson, 1991). Based on the distribution of the data, the Timed Up and Go scores were dichotomized into two categories: normal physical function ( ≤ 8.23 s) and preclinical limitations/limited physical function (>8.23 s), using cut points developed by Garber et al. (2010).

##### Thirty-Second Chair Stand

The 30-s chair stand measures the number of times a participant can rise from the chair in 30 s. Using a standard armless chair placed against the wall, and with their hands on the opposite shoulder, participants rise from a seated position to a standing position and return to a fully seated position as many times as possible in 30 s. The 30-s chair stand test has been validated for measuring lower body strength (*r* = 0.71) in community-dwelling older women (Rikli and Jones, 1999).

##### Thirty-Second Arm Curl

The 30-s arm curl measures the number of biceps curls that can be completed with the dominant arm in 30 s holding a 5 lbs (2.27 kg) hand weight for women or 8 lbs (3.63 kg) for men. The 30-s arm curl has been validated in measuring upper body strength in women (*r* = 0.79) (Rikli and Jones, 1999).

#### Level of Physical Activity

##### Stage of Change for Exercise

The stage of change for exercise was measured using an interviewer-administered questionnaire (Reed et al., 1997; Nigg and Riebe, 2002; Schumann et al., 2002). Exercise was defined as “planned physical activity performed to increase physical fitness completed three or more times per week for at least 20 minutes per session (Nigg and Riebe, 2002).” Participants were classified into one of five stages, based on their responses to the questionnaire. Participants classified as being in the precontemplation stage had no intention to begin exercising in the next 6 months, whereas those in the contemplation stage intended to begin exercising within the next 6 months. Individuals in the preparation stage intended to begin exercising within the next 30 days. Those in action had been exercising regularly for <6 months, and participants in maintenance had been exercising regularly for more than 6 months. Participants were then dichotomized into two groups [active group (AG) and inactive group (IG)] based on their stage of change (Riebe et al., 2009). Individuals in the action and maintenance stage were categorized as active because they reported engaging in at least 60 min of structured exercise per week, while individuals in the other stages were classified as inactive. To examine the influence of activity group and age on physical function performance, four activity-age groups were established (IG and under 80 years of age; IG and 80 years and older; AG and under 80 years of age; AG and 80 years and older).

#### Energy Expenditure

The Yale Physical Activity Scale (Dipietro et al., 1993) is an interviewer-administered survey that asks participants to estimate time spent in a list of 25 activities, ranging in intensity level, in a typical week during the last month. Weekly energy expenditure (kcal·week^−1^) was calculated by multiplying the time spent in each activity by an intensity code and then summed across all activities.

### Analyses

Data were examined for normality using skewness and kurtosis, and descriptive statistics were calculated for all key variables, with frequencies being determined for categorical variables and means and SD for continuous variables SPPS version 27 (IBM Corp., Armonk, NY) was utilized for the analyses and *p* < 0.05 was used to determine significance. Participants were included and retained in the SENIOR II study if they were able to provide data for most of the variables. In some cases, individuals were unable or unwilling to complete one or more assessments of physical function, resulting in missing data. Therefore, to conserve power, separate analyses were run for each of the outcome variables.

Independent samples *T*-tests and chi-square tests were conducted to determine if there were differences in the demographic characteristics between the AG and IG at baseline. Repeated measures ANOVA examined changes in each of the physical function measures (Timed Up and Go, 30-s chair stand, 30-s arm curl) from baseline to 48 months by activity group, with time as the within-subject factor and activity group being the between-subject factor. As age was significantly different between activity groups, an ANCOVA controlling for age then examined changes in each of the physical function measures as continuous variables from baseline to 48-months by activity group, with time as the within-subject factor and activity group being the between-subject factor. Chi-square analyses were used to examine the effect of activity group on Timed Up and Go categories (normal physical function vs. preclinical limitations/limited physical function) at 48-months. Lastly, chi-square analyses were used to examine the effect of the activity-age group and the Timed Up and Go categories at baseline and 48-months.

## Results

Table 1 shows baseline demographic information. The participants had a mean age of 79.06 ± 5.1 years and a mean BMI of 28.6 ± 5.6 kg/m^2^. The majority of participants were White (78.9%); other races/ethnicities included Portuguese (9.2%), Black (2.3%), and Cape Verdean (4.6%). The participants had a mean of 12.9 ± 2.4 years of education, with most participants (57.5%) having a high school diploma or less. Approximately 61% of the participants (*n* = 144) were classified as being in the IG at baseline, based on their stage of change for exercise. The IG was significantly older than AG (*p* = 0.005). There also was a significant difference in perceived health status (*p* = 0.008), with a greater proportion of AG than IG rating their health status as excellent or very good. There were no significant baseline group differences in smoking history or years of education (both *p* > 0.05).

**Table 1:** Demographic characteristics and physical function measures by activity group at baseline.

	**Sample** **(*n* = 238)**	**Range**	**Inactive** **group** **(*n* =144)**	**Active** **group** **(*n* =94)**	***P-*value**
**Demographics**	**Mean ±SD**		**Mean ±SD**	**Mean ±SD**	
Age (yr)	79.0 ± 5.1	67–94	79.7 ± 5.2	77.8 ± 4.7	0.005
Height (inches)	61.4 ± 3.37	47.3–75.3	61.2 ± 3.2	61.6 ± 3.4	0.410
Weight (lbs)	153.0 ± 30.2	74–340	156.7 ± 33.0	147.37 ± 24.3	0.020
BMI (m/kg^2^)	28.6 ± 5.6	18.6–67.0	29.4 ± 6.1	27.3 ± 4.3	0.002
Years of Education	12.9 ± 2.4	7–20	12.8 ± 2.3	13.2 ± 2.6	0.210
Energy Expenditure (Kcals/week)	7,015.0 ± 3,779.9	180–26,070	6,461.2 ± 3,692.1	7,863.3 ± 3,774.7	0.005
**Perceived** **health status**	***n*** **(%)**		***n*** **(%)**	***n*** **(%)**	
Excellent/very good	127 (53.4)		83 (38.8)	76 (55.1)	0.006
Good	90 (37.8)		96 (44.9)	50 (36.2)	
Fair/poor	21 (8.8)		35 (16.4)	12 (8.7)	

*Inactive group = precontemplation, contemplation, and preparation stages of change. Active group = action and maintenance stages of change. P-values were obtained using chi-square tests for categorical variables and t-test for continuous variables*.

Results of the repeated measures ANOVAs demonstrated a group × time interaction for Timed Up and Go performance, with the IG performing more poorly over time compared to the AG [F_(1, 199)_ = 4.48, *p* = 0.04]. There were no significant interactions for the 30-s arm curl or the 30-s chair stand. Main effects for activity group and time were present for 30-s arm curl performance {[F_(1, 187)_ = 12.02, *p* = < 0.001] and [F_(1, 187)_=13.64, *p* = < 0.001], respectively}. There was a main effect for activity group for the 30-s chair stand [F_(1, 175)_ = 12.62, *p* = <0.001].

When the data were analyzed using repeated measures ANCOVAs, controlling for age, there were no significant group by time interactions for the three measures of physical function. There was a significant main effect for activity group for the 30-s arm curl [F_(1, 186)_ = 7.32, *p* = 0.007] and the 30-s chair stand [F_(1, 174)_ = 10.06, *p* = 0.002] (See Table 2). Significant main effects for activity group and time were present for Timed Up and Go performance {[F_(1, 198)_ = 7.82, *p* = <0.006] and [F_(1, 198)_ = 5.85, *p* = <0.02], respectively}. There was a significant association between age and time for Timed Up and Go performance [F_(1, 198)_ = 7.02, *p* = 0.009], also shown in Table 2.

**Table 2:** Results of the repeated measures ANCOVA, controlling for age at baseline, examining physical function performance by activity groups at baseline and 48 months.

	**Activity group**				***P*-value** **(between groups)**	***P*-value** **(within subjects)**	**Interaction** **(group × time)**
	**Inactive group**		**Active group**				
	**Mean** **±SE**		**Mean** **±SE**				
	**BL**	**48**	**BL**	**48**			
Timed Up and Go (sec)^*^	11.0 ± 0.3	13.7 ± 0.8	9.6 ± 0.4	10.8 ± 1.0	0.006	0.016	0.149
30 s Chair Stand (reps)^∧^	10.7 ± 0.3	10.5 ± 0.3	12.2 ± 0.4	11.65 ± 0.3	0.002	0.162	0.387
30 s Arm Curl (reps)^#^	11.6 ±.31	12.4 ± 0.3	12.6 ± 0.4	13.6 ± 0.3	0.007	0.781	0.587

*ANCOVA analyses controlled for age at baseline (Age = 78.37 years)*.

*Inactive group = precontemplation, contemplation, and preparation stages of change. Active group = action and maintenance stages of change. Values presented as means ± SE*.

* *Timed Up and Go: Inactive Group, n = 118; Active Group, n = 83*.

∧ *30 s Chair Stand: Inactive Group, n = 100; Active Group, n = 77*.

# *30 s Arm Curl: Inactive Group, n = 111, Active Group, n = 78*.

**Figure 1:**
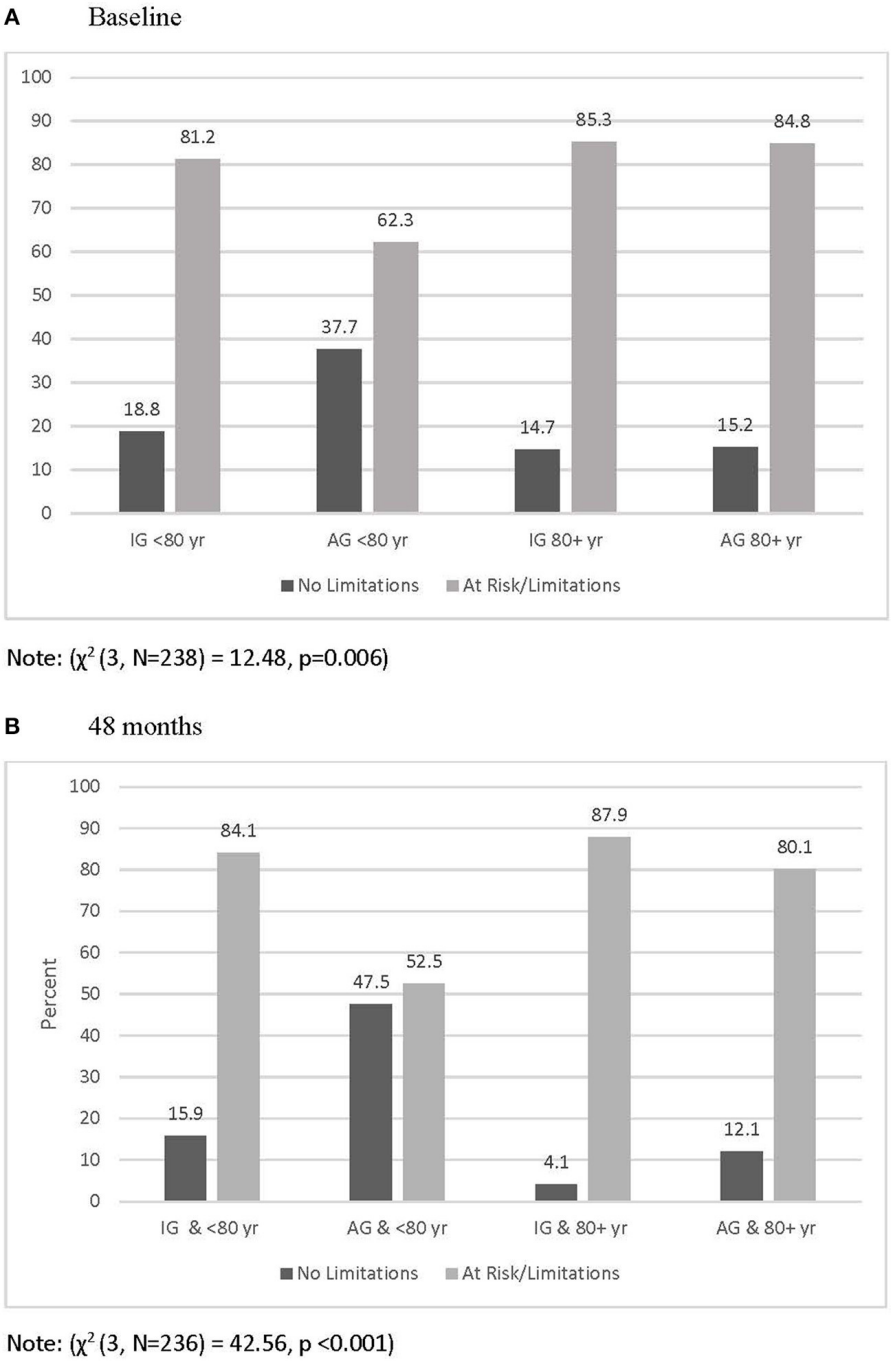
Percentage of participants classified as having no physical functional limitations and at risk or with limitation by age and activity group at baseline **(A)** and 48 months **(B)**.

The chi-square analyses indicated differential Timed Up and Go classifications between activity groups at the 48-month follow-up. As seen in Table 3, a greater percentage of individuals in the AG (35.1%) had normal levels of physical function compared to those in the IG (9.9%) [$\chi_{\left(1,\;\text{N}=236\right)}^{2}$ = 22.61, *p* < 0.001]. When Timed Up and Go classifications were examined by age and activity group) IG and under 80 years of age; IG and 80 years and older; AG and under 80 years of age; AG and 80 years and older), the chi-square analyses determined that activity-age group was associated with Timed Up and Go classification at baseline [$\chi_{\left(3,\;\text{N}=238\right)}^{2}$ = 12.48, *p* = 0.006] and 48-months [$\chi_{\left(3,\;\text{N}=236\right)}^{2}$ = 42.56, *p* < 0.001] (See Figure 1).

**Table 3:** TUG classification by activity group at 48 months.

	**Normal**	**Pre-clinical limitations or limitations**
	***N* (%)**	***N* (%)**
Active group	33 (35.1%)	61 (64.9%)
Inactive group	14 (9.9%)	128 (90.1%)

*[$\chi_{\left(1,\;\text{N}=236\right)}^{2}$ = 22.61, p < 0.001]*.

## Discussion

This study found that community-dwelling older women who self-reported engaging in regular exercise had better physical function performance compared to women who did not engage in exercise. The repeated measures ANCOVAs provided evidence of decreased physical function among both IG and AG from baseline to 48-months, though this change was only statistically significant for the Timed Up and Go. This finding agrees with other studies showing that increasing age is associated with poorer physical function (Kenny et al., 2013; Svinøy et al., 2021) but what is notable in the current study is that when controlling for age, there was no group x time interaction. However, since the AG had higher baseline levels of physical function, they remained more highly functioning compared to their IG counterparts at 48 months. Furthermore, a significantly larger proportion of the IG who were categorized as having preclinical physical function limitations or physical function limitations at 48-months (Garber et al., 2010).

The Timed Up and Go is a widely used performance measure of physical function that requires lower extremity strength, agility, gait, and dynamic balance (Podsiadlo and Richardson, 1991). Data consistently show that Timed Up and Go scores are highly predictive of several adverse health outcomes, including hospitalization, falls, mortality, low quality of life, and difficulties with ADLs (Olivares et al., 2011; Viccaro et al., 2011; Ascencio et al., 2022). Cross-sectional data from the population based Norwegian Tromsø Study found Timed Up and Go scores increased by 0.14 s per year in women aged 65–84 years (Svinøy et al., 2021). In the current study, the time needed to complete the Timed Up and Go increased 1.2 s for the AG over 48-months, or on average 0.3 s per year, while the increase experienced by the IG was over two times higher during this same period (2.7 s increase over 48-months, 0.68 s per year), when the data was adjusted for age.

It is not surprising that the IG was more likely than AG to have Timed Up and Go scores indicating pre-clinical physical function limitations or physical function limitations due to the influence of physical activity on physical function performance over the lifespan (Harridge and Lazarus, 2017). There was evidence for decreases in physical function for participants who were <80 and 80+ years, which has been demonstrated in other longitudinal studies (Leigh et al., 2017; Dipietro et al., 2019). However, regardless of age group, a greater percentage of the IG had Timed Up and Go scores that were classified as being at risk or indicative of limitations at 48-months. In the current study, physical activity appeared to be more protective in the younger vs. the older group both at baseline and at 48-months.

Interestingly, the arm curl test scores across the 4-year follow-up period increased for both groups, indicating improved upper body physical function. This finding is noteworthy, as prior research suggests that physical function declines with age beginning at midlife (Kuh et al., 2005) and that lower handgrip strength to body mass ratio is associated with increased odds for poorer physical function (Barbat-Artigas et al., 2012). It is, however, possible that women compensated for declines in lower body strength by increased reliance on their upper body, which contributed to increased strength. It has been shown that when preclinical signs of physical function limitation emerge, individuals resort to compensatory strategies to perform everyday activities, such as pushing with their upper body, using the chair arms or their thighs, to assist them in standing from a seated position (Von Bonsdorff and Rantanen, 2011).

The stage of change questionnaire was used to determine exercise behavior (Riebe et al., 2009). The questionnaire was administered by trained research assistants in this study, but the measure can also be self-administered. Our findings suggest that participants who were classified as being in the action and maintenance stage at baseline had better Timed Up and Go scores at baseline and at 48 months, suggesting that self-reported stage of change was able to differentiate between active and inactive women. However, it should be noted that the stage of change for exercise questionnaire is a self-report measure, and as has been well-documented self report physical activity does not always agree with objectively measured physical activity (Ogonowska-Slodownik et al., 2021). Energy expenditure (kcals·wk^−1^) data from the Yale Physical Activity Scale confirms that physical activity was significantly different between the activity groups at baseline, but it should be noted that this is also a self-report measure. Studies utilizing cohorts of community-dwelling older women may want to consider adding a stage of change measure for exercise to their methodology due to its ease of use. More importantly, health care providers should consider administering the stage of change algorithm as a simple and effective method of assessing physical activity level as part of routine appointments. Individuals classified as being inactive (contemplation, contemplation, and preparation stages) could be offered health education materials or intervention opportunities that align with their stage of change for physical activity and exercise.

At baseline, self-reported health differed between activity groups, with those in the AG more likely to report their health as being good or excellent compared to the IG. This is consistent with the work of Lohne-Seiler et al. (2014) who found that physical activity levels measured by accelerometry differed across categories of self-reported health, with those who reported very good health having 51% higher counts per minute compared to those with poor or very poor health. Although we found an association between physical activity level and perceived health, due to the study design, we cannot determine causality. Those with poor or declining health may be less likely to be physically active; however, lower levels of physical activity may lessen conditioning, which in turn negatively affects health.

In the U.S., life expectancy is longer for women than men, but women are more likely than men to develop debilitating conditions and often live longer with disability (Freedman et al., 2016). Studies, including this study, have consistently found that physical activity is associated with better physical function in older women in clinical and community-dwelling populations (Yorston et al., 2012; Chmelo et al., 2013; Walker et al., 2021). Unfortunately, physical activity declines with age, and older women are the least active group of all age groups as only 12% of women ≥ 65 years meet national guidelines for aerobic and muscle strengthening activities (Federal Interagency Forum on Aging-Related Statistics, 2020).

Because this was a longitudinal community-based study with older adults, we anticipated that some participants would drop out of the study while others would be unable to complete all the physical assessments. To allow for the inclusion of as many participants as possible, participants with baseline and 48-month data for at least one variable of interest were included in our analyses. We examined missing data for objectively measured physical function assessments, and determined that those in the IG were more likely to have missing data compared to the AG (Timed Up and Go, 18.1 vs. 11.7%; 30-s chair stand, 30.6 vs. 18.1%; 30-s arm curl 17.0 vs. 22.9%, respectively). Therefore, it is unlikely that the pattern of missing data was random, as respondents self selected not to complete physical function assessments. This suggests that individuals in the IG were less healthy and had more limitations compared to the AG. If all study participants had completed the assessments, it is possible that the differences between the groups would have been greater. Due to the amount and non-random nature of the missing data, we provided the *N* for each physical function assessment in Table 2.

Study findings should be considered with respect to study limitations, which include a study population that was predominately White. Study participants may have a greater interest in health and wellbeing as they previously enrolled in a community-based health promotion study, which could limit the generalizability of study findings. Additionally, the use of the stages of change to classify participants as active and inactive does not indicate if they meet the recommendations for aerobic or resistance training as indicated by the 2018 Physical Activity Guidelines for Americans (U.S. Department of Health and Human Services, 2018). As expected, there was a significant amount of missing data, particularly in the IG. While the current study used listwise deletion to handle missing data, some research suggests that multiple imputation may have produced less biased results (van Ginkel et al., 2020). Finally, we did not include a measure of frailty in this study. Previous research has identified an association between increasing age and frailty with declines in physical activity and physical function (Gil-Salcedo et al., 2020), in addition to poor health outcomes (Vermeiren et al., 2016) and increased risk for falls (Kojima, 2015). Future studies examining physical function and older adults should account for this association.

Study strengths include using several well-established objective measures of physical function, and a long follow-up period (48-months). Additionally, it should be noted that data were collected in the community at participants' homes by trained members of their peer group. This approach could allow for research teams to access participants more readily in rural communities, especially as technology makes remote training of research associates a possibility.

In conclusion, we found that community-dwelling older women who reported engaging in regular exercise at baseline had higher levels of objectively measured physical function and were less likely to have Timed Up and Go scores that indicated pre-clinical physical function limitations or physical function limitations compared to those who were inactive. Aging has a deleterious effect on physical function performance. In this study while exercise did not prevent declines in performance over time, those who were the most active initially outperformed those who were inactive at baseline and 4 years later. These findings add to the body of literature that support the long-term benefits of exercise on physical function in older women.

## Data Availability Statement

All reasonable requests for the datasets analyzed for this study can be made to the corresponding author.

## Ethics Statement

The studies involving human participants were reviewed and approved by University of Rhode Island, Institutional Review Board. The patients/participants provided their written informed consent to participate in this study.

## Author Contributions

CW-R and DR contributed to conceptualization and design of the study. CW-R, DR, MG, and PC contributed to data analysis and interpretation, in addition to manuscript writing and revision. The submitted version has been read and approved by all authors.

## Funding

This work was supported by Grant 5RO1CA126448 from the National Cancer Institute (NCI) and the National Institute on Aging (NIA), National Institutes of Health, to the University of Rhode Island (PI: PC). Neither NCI nor NIA had any direct involvement in the study design; the collection, analysis and interpretation of data; the writing of the report; and the decision to submit this article for publication.

## Conflict of Interest

The authors declare that the research was conducted in the absence of any commercial or financial relationships that could be construed as a potential conflict of interest.

## Publisher's Note

All claims expressed in this article are solely those of the authors and do not necessarily represent those of their affiliated organizations, or those of the publisher, the editors and the reviewers. Any product that may be evaluated in this article, or claim that may be made by its manufacturer, is not guaranteed or endorsed by the publisher.
